# P-1967. Characteristics of the first immunocompromised patients to receive Sipavibart as an early access treatment for COVID-19 pre-exposure prophylaxis in France

**DOI:** 10.1093/ofid/ofae631.2126

**Published:** 2025-01-29

**Authors:** Paul Loubet, Benjamin Gaborit, Mathilde Salpin, Hélène Gardeney, Iliès Benotmane, Thomas Systchenko

**Affiliations:** CHU de Nîmes, Nimes, Languedoc-Roussillon, France; CHU de Nantes, Nantes, Pays de la Loire, France; CHU Bichat, Paris, Paris, Ile-de-France, France; CHU de Poitiers, Poitiers, Centre, France; CHU de Strasbourg, Strasbourg, Alsace, France; CHU de Poitiers, Poitiers, Centre, France

## Abstract

**Background:**

Immunocompromised patients account for a large proportion of COVID-19 hospitalizations and deaths, even in the most recent periods, despite having received several COVID-19 vaccine doses. Monoclonal antibodies effectively prevented COVID-19 in the general population in randomized control trials and the immunocompromised population in real-world studies. Unfortunately, the emergence of new variants precluded their use. Sipavibart (AZD3152) is an investigational long-acting monoclonal antibody designed to provide broad coverage across Omicron and ancestral viral variants currently being studied in the SUPERNOVA prevention trial. France was the first country to grant Sipavibart as a COVID-19 pre-exposure prophylaxis treatment in immunocompromised individuals with an early access authorization in December 2023.

**Figure:**
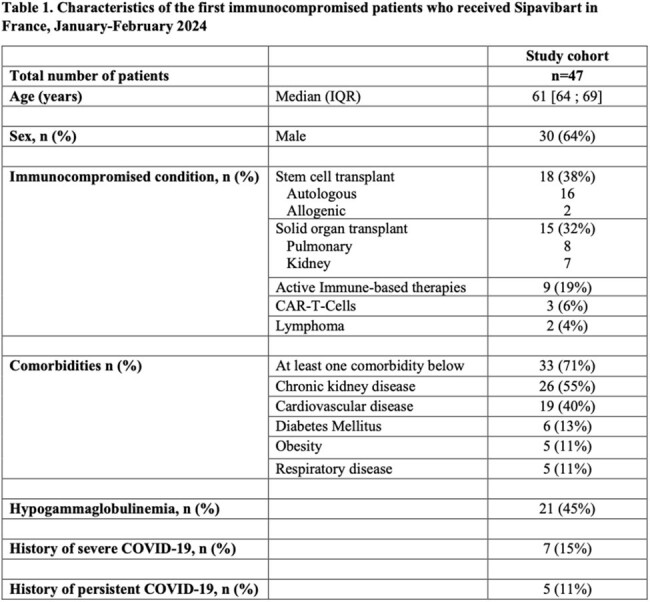

**Methods:**

We performed a retrospective multicentric study in France, including the first consecutive patients to receive one intramuscular dose of Sipavibart in January and February 2024, to collect their characteristics and COVID-19-related medical history.

**Results:**

Overall, we included 47 patients with a median age of 61 years (IQR:64—69) and 64% male. The three main immunosuppressive conditions were Stem cell transplants (38%), solid organ transplants (32%), and immune-based therapies (mostly Rituximab) (19%) for chronic inflammatory diseases or haematologic malignancies. Nearly half had hypogammaglobulinemia and 71% had at least another key comorbidity (Table 1). 15% and 11% had a history of severe and persistent COVID-19, respectively. The median number of COVID-19 doses was 4 (IQR 3-6), and nearly one-third had previously received previous generation of monoclonal antibodies for COVID-19 prophylaxis (Casirivimab/imdevimab or Tixagevimab/Cilgavimab). SARS-CoV-2 serology results before Sipavibart administration were available in 11 patients (without Immunoglobulins substitution). The median anti-Spike IgG titers was 30 UI/L (IQR 0-179).

**Conclusion:**

The first patients to receive Sipavibart in France had different profiles, but they were all highly immunocompromised with frequently associated hypogammaglobulinemia and other chronic conditions.

**Disclosures:**

Paul LOUBET, MD, PhD, Astrazeneca: Advisor/Consultant|Gilead: Advisor/Consultant|Moderna: Advisor/Consultant|Pfizer: Advisor/Consultant Iliès Benotmane, n/a, Astrazeneca: Advisor/Consultant|Astrazeneca: Board Member

